# Allergic and immunologic evaluation of children with celiac disease

**DOI:** 10.3389/fped.2025.1568174

**Published:** 2025-04-09

**Authors:** Duygu Demirtaş Güner, Kübra Baskın

**Affiliations:** ^1^ Department of Pediatric Gastroenterology, Hepatology and Nutrition, Van Training and Research Hospital, Van, Türkiye; ^2^ Department of Pediatric Allergy and Immunology, Van Training and Research Hospital, Van, Türkiye

**Keywords:** allergy, antibody deficiency, celiac disease, children, immunodeficiency, immune dysregulation

## Abstract

**Introduction:**

Celiac disease (CD) and allergic diseases are immune-mediated disorders with overlapping clinical and immunologic features. The association between CD and selective immunoglobulin (Ig) A deficiency (sIgAD) is well-established, but limited data exist on the relationship between CD, other antibody deficiencies, and allergic diseases in children. This study aimed to evaluate the prevalence of allergic manifestations and immunologic abnormalities in children with CD.

**Methods:**

This prospective study included children with biopsy-confirmed CD, followed at a gastroenterology clinic from August 2022 to February 2023. Participants underwent comprehensive immunologic and allergic evaluation, including serum immunoglobulin levels, vaccine antibody responses, lymphocyte subgroup analysis, and allergy testing as clinically indicated.

**Results:**

The cohort included 76 patients with a median age of 11 years and a median age at CD diagnosis of 5.8 years. Allergic manifestations included aeroallergen sensitivity (22.4%), allergic rhinitis (15.8%), allergic conjunctivitis (13.2%), food allergy (5.3%), and asthma and eczema (3.9% each). Immunologic evaluations revealed normal profiles in 69.7% of patients, while abnormalities included partial IgM deficiency (6.6%), unclassified hypogammaglobulinemia (5.3%), sIgAD (2.6%), and transient hypogammaglobulinemia of infancy (2.6%). Elevated IgE levels were observed in 13.2% of patients.

**Conclusion:**

This study highlighted a significant prevalence of allergic diseases and immunologic abnormalities in children with CD, extending beyond the commonly recognized association with sIgAD. These findings underscore the importance of comprehensive immunologic and allergic evaluation in children with CD.

## Introduction

Celiac disease (CD) is an immune-mediated enteropathy triggered by dietary gluten in genetically predisposed individuals. It affects 0.7%–1.4% of the population worldwide, with a higher prevalence in pediatric populations compared to adults (1).

The cornerstone of CD diagnosis relies on tissue transglutaminase immunoglobulin (Ig) A (TGA-IgA) testing, typically accompanied by total IgA measurement to identify selective IgA deficiency (sIgAD). This practice emerged from the well-documented association between CD and sIgAD, which occurs more frequently in patients with CD than in the general population (2–4). As a result, TGA-IgA levels may be low in patients with CD who also have sIgAD, which can complicate diagnosis. In cases of sIgAD, alternative testing using IgG-based assays becomes essential for accurate diagnosis. It is recommended to check for deamidated gliadin peptide IgG, tissue transglutaminase IgG, or anti-endomysial IgG in children with low total IgA levels (2).

Consequently, pediatric gastroenterologists routinely measure total IgA levels alongside TGA-IgA during screening or in suspected CD cases. However, other immunoglobulin levels are often overlooked, potentially leaving immunodeficiencies undiagnosed. For instance, common variable immunodeficiency (CVID) can present with gastrointestinal manifestations mimicking CD, including villous atrophy. Without comprehensive immunoglobulin evaluation, such cases might be misdiagnosed as seronegative CD (5, 6).

Beyond immunodeficiencies, recent evidence suggests that allergic conditions, including atopic dermatitis, allergic rhinitis, asthma, and food allergies, have an increased prevalence in patients with CD. This association suggests shared immune dysregulation mechanisms in CD and allergic diseases (7–10).

There is growing interest in understanding the potential relationships between CD, immunodeficiencies, and allergic diseases. While early identification of coexisting conditions can guide comprehensive management strategies, further studies are needed to clarify these associations (5, 9, 11).

This study aimed to characterize the immunologic and allergic profiles of children with CD, with particular attention to immunoglobulin levels, allergic sensitization, and immunodeficiencies beyond sIgAD.

## Materials and methods

### Study design and population

This prospective cohort study was conducted at the pediatric gastroenterology clinic of Van Training and Research Hospital between August 21, 2022, and February 21, 2023. Children with biopsy-confirmed CD, diagnosed according to the Marsh-Oberhuber classification criteria, were enrolled.

Inclusion criteria were as follows: (a) Children aged 2–18 years. (b) Biopsy-confirmed CD diagnosis. (c) Informed consent obtained from parents/guardians.

Exclusion criteria were as follows: (a) Patients undergoing immunosuppressive therapy. (b) Patients with incomplete clinical or laboratory data. (c) Presence of other chronic diseases affecting immune function (e.g., primary immunodeficiencies)*.* (d) Inability to complete study procedures.

### Clinical and laboratory evaluation

Patients were referred to the pediatric allergy and immunology clinic for a comprehensive evaluation as part of the study protocol. Serum immunoglobulin levels (IgA, IgM, IgG, IgE) were measured using turbidimetric methods, with age-specific reference ranges applied. For patients with low immunoglobulin levels, a second measurement was performed at least one month later to confirm the diagnosis before classification. Patients with persistently low or abnormal immunoglobulin levels underwent further immunologic evaluation, including lymphocyte subset analysis (CD3+, CD4+, CD8+, CD19+, and CD16+/56+) (12). Vaccine antibody responses (anti-HBs and anti-rubella IgG) were assessed to evaluate immune function.

Patients were evaluated for gastrointestinal and allergic symptoms, including dysphagia and food impaction. However, routine endoscopic screening for eosinophilic esophagitis (EoE) was not performed.

Allergy testing included skin prick tests (SPTs) for common aeroallergens (e.g., pollen, house dust mites, grass pollens, molds, animal dander) and food allergens (e.g., milk, eggs, wheat). SPTs were performed using standardized allergen extracts, and a wheal diameter ≥3 mm greater than that of the negative control was considered positive. Respiratory function testing was performed for patients with respiratory symptoms.

### Statistical analysis

Data were analyzed using SPSS for Windows, version 22.0. Continuous variables were expressed as medians with interquartile ranges (IQR), and categorical variables as counts and percentages. The normality of continuous variables was assessed using the Shapiro–Wilk test**.** Based on this assessment, appropriate statistical methods were applied: normally distributed data were analyzed using the Student's *t*-test, while non-normally distributed data were analyzed using the Mann–Whitney *U*-test. A *p*-value < 0.05 was considered statistically significant.

The study was approved by the Institutional Review Board of Van Training and Research Hospital (decision number: 2023/13–06), and written informed consent was obtained from all participants’ parents.

## Results

### Demographic and clinical characteristics

The study included 76 children diagnosed with CD [52 females (68.4%), 24 males (31.6%)], with a median age of 11 years (IQR 7.2–14.3) and a median age at diagnosis of 5.8 years (IQR 3.8–9.8). Parental consanguinity was present in 24 patients (31.6%), and 13 patients (17.1%) reported family history of atopy. During the study, 27 patients (35.5%) had negative TGA-IgA results, indicating that these patients had been adhering to a gluten-free diet (GFD), leading to serological normalization. In 28 patients (36.8%), TGA-IgA was positive but less than 10 times the upper limit of normal (ULN). In 19 patients (25%), TGA-IgA was greater than 10 times the ULN (≥200 RU/ml). Tissue transglutaminase IgA levels were missing in two patients.

None of the patients had symptoms suggestive of EoE, such as dysphagia or food impaction. Therefore, no additional endoscopic evaluation for EoE was performed. However, asymptomatic cases of EoE may have gone undetected.

### Allergic manifestations

Allergic diseases were prevalent among the patients. Allergic rhinitis was diagnosed in 15.8% of the cohort, while allergic conjunctivitis was observed in 13.2%. Food allergy or a history of food allergy was present in 5.3% of the patients, and asthma and eczema were diagnosed in 3.9% of the patients each. These findings are summarized in Table 1. Of the four patients with food allergy, one was allergic to milk, eggs, walnuts, and sesame; another had a history of egg and lamb allergy; a third had a history of milk and egg allergy; and the last patient had a history of peanut allergy.

**Table 1:** Prevalence of allergic diseases accompanying celiac disease.

Allergic disease	n (%)
Allergic rhinitis	12 (15.8%)
Allergic conjunctivitis	10 (13.2%)
Food allergy	4 (5.3%)
Asthma	3 (3.9%)
Eczema	3 (3.9%)

### Immunological findings

None of the patients had neutropenia or lymphopenia. Normal immunoglobulin levels and antibody responses were observed in 69.7% of the patients, while immunological abnormalities were identified in 30.3%. The most common abnormality was partial IgM deficiency, followed by unclassified hypogammaglobulinemia, sIgA deficiency, and a preliminary diagnosis of transient hypogammaglobulinemia of infancy. The latter was diagnosed in two patients (aged 2 and 4 years) based on low IgG levels, normal vaccine responses, absence of growth failure, and no evidence of T-cell defects, according to the clinical criteria of the European Society for Immunodeficiencies (ESID) Registry (13). None of the patients had a history of frequent infections. Elevated total IgE levels were observed in 13.2% of the patients.

**Figure 1:**
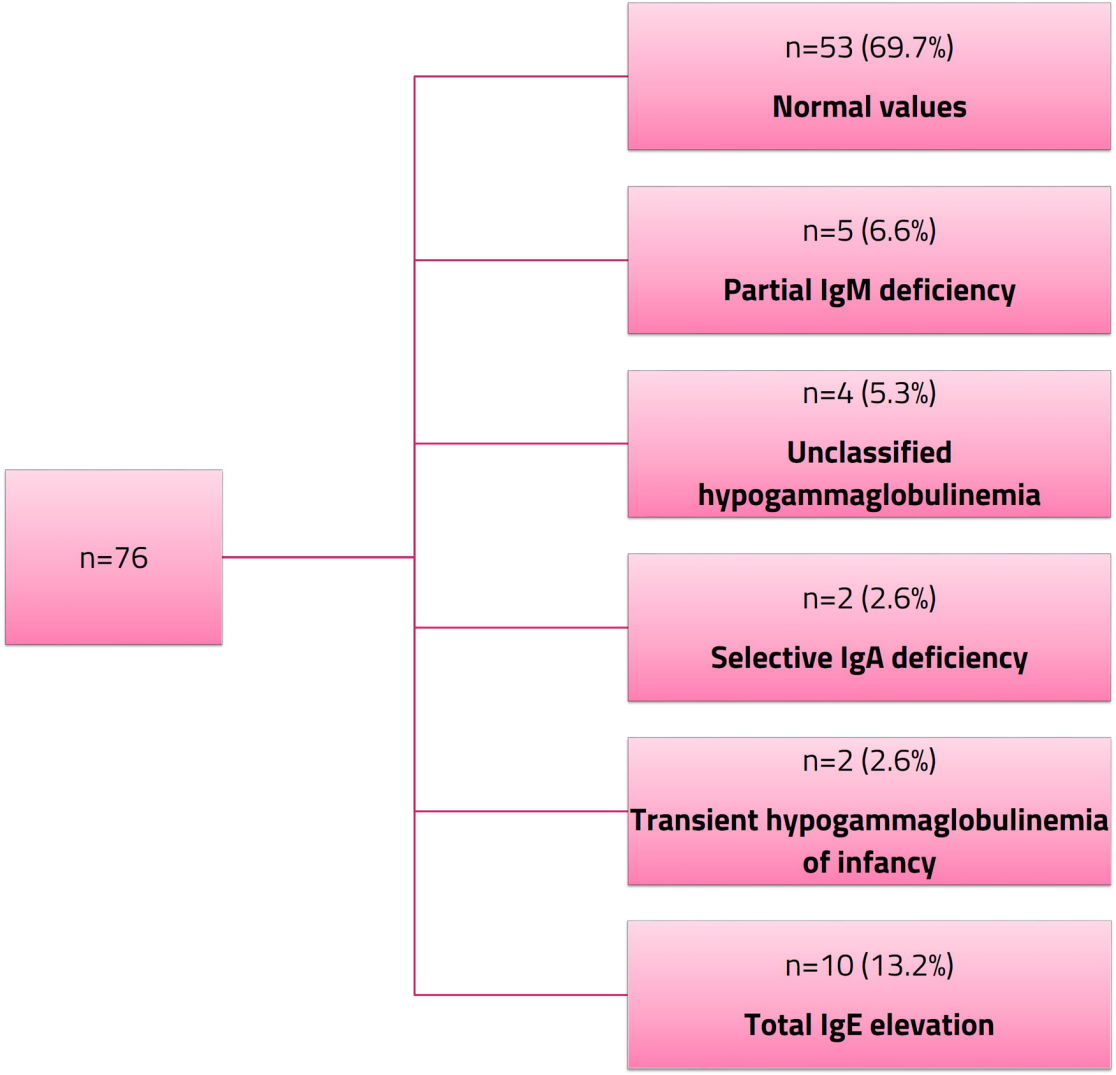
Immunological abnormalities in pediatric celiac disease.

Further details on the distribution of immunological abnormalities are summarized in Figure 1.

The four patients with unclassified hypogammaglobulinemia underwent immunologic evaluation with simultaneous measurement of TGA-IgA levels at a median of 5.6 years after CD diagnosis. Two of these patients had negative TGA-IgA levels and were adherent to a GFD, while the other two had persistently elevated TGA-IgA levels due to nonadherence. Similarly, among patients with partial IgM deficiency, three had elevated TGA-IgA levels due to nonadherence to a GFD.

## Discussion

Our study highlights the association between CD, allergic diseases, and immunologic abnormalities in children. We identified allergic diseases such as allergic rhinitis, allergic conjunctivitis, food allergy, asthma, along with immunologic abnormalities including partial IgM deficiency, unclassified hypogammaglobulinemia, sIgAD, and a preliminary diagnosis of transient hypogammaglobulinemia of infancy. Importantly, these immunologic abnormalities extend beyond the commonly recognized sIgAD associated with CD, suggesting a broader spectrum of immune dysregulation may coexist with CD and contribute to immune dysfunction.

The link between CD and allergic diseases has been increasingly recognized, with studies suggesting that children with allergic rhinitis, allergic conjunctivitis, asthma, and atopic eczema have a higher risk of developing CD compared to the general population. One study demonstrated adjusted incidence rate ratios of 1.39 for allergic rhinitis/conjunctivitis, 1.44 for atopic eczema, and 1.41 for asthma in adults, highlighting the importance of considering CD screening in patients presenting with allergic symptoms (14). Our findings are consistent with previous studies and further support the clinical association between CD and allergic diseases. In our cohort, 22.4% of patients exhibited aeroallergen sensitivity, 15.8% had allergic rhinitis, 13.2% had allergic conjunctivitis, 3.9% had asthma, and 3.9% had eczema. Additionally, elevated IgE levels were observed in 13.2% of our patients, particularly among those with allergic conditions such as eczema and asthma, supporting the hypothesis of immune dysregulation in CD. A study involving 2,297 individuals found a higher prevalence of IgE sensitization to specific allergens such as wheat, house dust mites, and food allergens in patients with CD (9).

IgE-mediated food hypersensitivity is relatively common among children with CD. One study reported that 50% of children with CD had a positive SPT for at least one food allergen, with peanuts being the most commonly identified sensitization (8). In our cohort, food allergy was documented in 5.3% of patients, with allergen profiles indicating sensitivity to milk, egg, nuts, sesame, and meat (lamb).

The relationship between CD and severe food allergy, defined as high IgE levels combined with a history of severe allergic reactions, has been investigated. One study found that children with severe food allergy had a higher prevalence of CD compared to those with mild allergy or the general population, particularly in the presence of elevated IgE levels (15). Although severe food allergy was not identified in our study, the elevated IgE levels in patients with allergic diseases underscores the complex interplay between CD and allergic sensitization. These findings highlight the importance of screening for CD in children with allergic profiles, even in the absence of severe food allergies. Similarly, screening for allergies in children diagnosed with CD is essential to ensure comprehensive management and address any coexisting allergic conditions.

Increased intestinal permeability in CD, which may increase antigen exposure and promote subsequent allergic sensitization, has been proposed as a potential mechanism underlying the overlap between celiac disease and allergic diseases (9, 15). Additionally, mast cells have been implicated in CD pathogenesis, suggesting another potential mechanism that warrants further investigation (16). Although our study did not directly evaluate these mechanisms, future research is needed to better understand the immunologic interplay between CD and allergic diseases.

It is important to note that immunodeficiencies such as CVID, sIgAD, and IgM deficiencies can present with gastrointestinal symptoms that mimic those of CD, thereby complicating its diagnosis. When coexisting with CD, these immunodeficiencies may lead to false-negative serological results due to insufficient immunoglobulin production (5). This underscores the need for pediatric gastroenterologists to extend their immunological assessments beyond IgA levels, particularly in patients with refractory symptoms despite adherence to a GFD.

Adherence to a GFD is crucial for intestinal mucosal healing, which facilitates the normalization of immunoglobulin levels by improving nutrient absorption and supporting immune function (17, 18). However, persistent immunoglobulin abnormalities despite strict GFD adherence may indicate the presence of primary immunodeficiencies, such as selective IgM deficiency (sIgMD), which require ongoing monitoring and treatment (19).

In our study, partial IgM deficiency and unclassified hypogammaglobulinemia were identified in 11.8% of patients, suggesting a potential role in immune dysregulation in CD. Notably, these abnormalities were observed in both GFD-adherent and non-adherent patients, suggesting that immune dysfunction in CD may not be solely attributed to gluten exposure or intestinal inflammation but may also involve an underlying immunologic component. Non-adherent patients with persistently elevated TGA-IgA levels may develop secondary hypogammaglobulinemia due to ongoing intestinal inflammation. However, the persistence of immunoglobulin deficiencies despite strict dietary adherence suggests an underlying primary immunodeficiency, warranting further investigation. Regular monitoring of dietary adherence is essential in patients with CD, particularly those with coexisting immunologic abnormalities, as continued gluten exposure may exacerbate both gastrointestinal symptoms and immune dysfunction.

Even with strict adherence to a GFD, some patients with CD may continue to experience gastrointestinal symptoms due to coexisting food allergies. For instance, a case report described a 13.5-year-old girl with persistent symptoms despite strict GFD adherence who was diagnosed with a non-IgE-mediated soy allergy that resolved following soy elimination (20). This case highlights the need to consider additional food allergies in children with CD who have refractory symptoms. In our cohort, 5.3% of patients had documented food allergies, emphasizing the necessity of comprehensive allergen screening and management to optimize outcomes.

This study had several strengths that enhance the validity and reliability of our findings. First, its prospective design enabled systematic and standardized data collection. Second, the inclusion of only biopsy-confirmed CD cases ensured diagnostic accuracy. Third, the comprehensive immunological evaluation, including the assessment of all immunoglobulin classes and vaccine responses, provides novel insights beyond the typical focus on IgA levels. Additionally, the use of both clinical assessments and objective testing for allergic conditions strengthens the reliability of our findings.

Despite some limitations, including being conducted at a single center with a relatively small sample size, our results may have limited generalizability to the broader pediatric CD population. Moreover, the absence of a control group restricted our ability to compare the prevalence of immunologic and allergic abnormalities with the general population. Additionally, due to the short study duration, we were unable to reassess immunoglobulin levels after TGA-IgA normalization in some patients, leaving uncertainty about whether these abnormalities resolve with strict dietary adherence.

In conclusion, our findings suggested that CD may be part of a broader spectrum of immune dysregulation, encompassing allergic and immunologic components. The overlap between CD, allergic diseases, and immunologic abnormalities highlighted the importance of comprehensive immunological and allergic evaluations in children with CD, ensuring early detection and management to optimize care and improve outcomes.

Our study underscored the need for an integrated, multidisciplinary approach to managing children with CD, addressing the complex interplay of immune dysregulation and allergic manifestations associated with this condition.

## Data Availability

The original contributions presented in the study are included in the article/Supplementary Material, further inquiries can be directed to the corresponding author.
